# Acute stroke and TIA patients have specific polygraphic features of obstructive sleep apnea

**DOI:** 10.1007/s11325-019-02010-2

**Published:** 2020-01-14

**Authors:** Akseli Leino, Susanna Westeren-Punnonen, Juha Töyräs, Sami Myllymaa, Timo Leppänen, Salla Ylä-Herttuala, Anu Muraja-Murro, Anne-Mari Kantanen, Jaana Autere, Pekka Jäkälä, Esa Mervaala, Katja Myllymaa

**Affiliations:** 1 Department of Clinical Neurophysiology, Diagnostic Imaging Center, Kuopio University Hospital, Kuopio, Finland; 2 Department of Applied Physics, University of Eastern Finland, Kuopio, Finland; 3 School of Information Technology and Electrical Engineering, The University of Queensland, Brisbane, Australia; 4 Department of Neurology, NeuroCenter, Kuopio University Hospital, Kuopio, Finland; 5 Department of Neurology, University of Eastern Finland, Kuopio, Finland; 6 Department of Clinical Neurophysiology, University of Eastern Finland, Kuopio, Finland

**Keywords:** Sleep apnea, Stroke, Transient ischemic attack, Apnea-hypopnea index, Severity estimation

## Abstract

**Purpose:**

Obstructive sleep apnea (OSA) is associated with increased risk for stroke, which is known to further impair respiratory functions. However, it is unknown whether the type and severity of respiratory events are linked to stroke or transient ischemic attack (TIA). Thus, we investigate whether the characteristics of individual respiratory events differ between patients experiencing TIA or acute ischemic stroke and matched patients with clinically suspected sleep-disordered breathing.

**Methods:**

Polygraphic data of 77 in-patients with acute ischemic stroke (*n* = 49) or TIA (*n* = 28) were compared to age, gender, and BMI-matched patients with suspected sleep-disordered breathing and no cerebrovascular disease. Along with conventional diagnostic parameters (e.g., apnea-hypopnea index), durations and severities of individual apneas, hypopneas and desaturations were compared between the groups separately for ischemic stroke and TIA patients.

**Results:**

Stroke and TIA patients had significantly shorter apneas and hypopneas (*p* < 0.001) compared to matched reference patients. Furthermore, stroke patients had more central apnea events (*p* = 0.007) and a trend for higher apnea/hypopnea number ratios (*p* = 0.091). The prevalence of OSA (apnea-hypopnea index ≥ 5) was 90% in acute stroke patients and 79% in transient ischemic attack patients.

**Conclusion:**

Stroke patients had different characteristics of respiratory events, i.e., their polygraphic phenotype of OSA differs compared to matched reference patients. The observed differences in polygraphic features might indicate that stroke and TIA patients suffer from OSA phenotype recently associated with increased cardiovascular mortality. Therefore, optimal diagnostics and treatment require routine OSA screening in patients with acute cerebrovascular disease, even without previous suspicion of OSA.

**Electronic supplementary material:**

The online version of this article (10.1007/s11325-019-02010-2) contains supplementary material, which is available to authorized users.

## Introduction

Obstructive sleep apnea (OSA) is associated with an increased risk of ischemic stroke [1] and transient ischemic attack (TIA) [2]. The prevalence of OSA (apnea-hypopnea index (AHI) over 5 events/h) is estimated to be 70.4% in stroke and TIA patients and 39.5% of those have AHI over 20 events/h [3]. Untreated OSA worsens the outcomes of stroke rehabilitation and increases the risk for recurrent cardiovascular events [4–6]. Enhancing the diagnostics of OSA and improving the undestanding of the relationship between stroke, TIA and OSA could help to prevent recurrent cardiovascular events and have a major impact on decreasing the economic burden of stroke [4–6].

Currently, the diagnostics of OSA is primarily based on AHI [7], which ignores the durations of the respiratory events and the severity of the related oxygen desaturation events. A recent study showed an association between short respiratory events and all-cause mortality [8], which might connect short events to lower arousal threshold and elevated sympathetic tone. In addition, novel diagnostic parameters incorporating the severity of the desaturations were introduced [9–11] and they were reported to predict the severe health consequences of OSA better than AHI [11, 12]. All these studies indicate that patients with similar AHI may experience significantly different physiological stress and hemodynamic oscillations caused by the apneas and hypopneas.

In the current clinical practice, routine screening for sleep-disordered breathing is uncommon in patients with acute cerebrovascular disease. The aim of this study is to compare the disease of suspected sleep-disordered breathing patients, i.e., patients who already get treatment if needed, to those of acute cerebrovascular disease patients. We evaluated whether patients suffering from acute ischemic stroke or TIA with no previous diagnosis of OSA have different characteristics of individual obstruction and desaturation events compared to age, gender, and body mass index (BMI) matched patients with a clinical suspicion of OSA but no history of cerebrovascular disease. We hypothesized that patients with cerebrovascular disease would have longer apneas and hypopneas and more severe desaturations.

## Patients and methods

Seventy-nine patients with a first episode of acute cerebrovascular disease were recruited into the study. Patients were chosen and recruited by experienced neurologists from the neurological ward of the Kuopio University Hospital during 2015–2017. Exclusion criteria for recruitment of stroke patients were extensive brainstem ischemia, malignant middle cerebral artery infarction, severe stroke in cerebrum, clinically significant cognitive problems, or continuous positive airway pressure (CPAP) treatment. TIA was defined in accordance with the scientific statement by American Heart Association (AHA) [13]. Polygraphy recording supplemented with frontal electroencephalography (EEG) registration [14, 15] was conducted in patients recovering from TIA or mild-to-moderate ischemic stroke in the neurological ward of the Kuopio University Hospital (KUH). A single-night recording was conducted within 4 days of the onset of the stroke or TIA. The Research Ethics Committee of Hospital District of Northern Savo, Kuopio, Finland, gave a favorable opinion for the data collection (50/2014). Verbal and written information about the study protocol was given to the patients and informed consent was acquired from all patients or their next of kin prior to polygraphy recording. One patient was excluded from the study due to a change in diagnosis, and one patient due to a technically failed recording. Thus, a total of 77 patients, 49 with an ischemic stroke and 28 with a TIA diagnosis were ultimately included in the study. Each of these patients was matched (age, gender, BMI) with one patient with suspected OSA but no cerebrovascular disease. The reference patients were sought from a large set of patients (*n* > 400) referred to the KUH because of suspected sleep-disordered breathing during the years 2002–2014. The matching of the patients was performed by first matching the gender and then minimizing a comparison value (CV) calculated using the Eq. 1, where *C* denotes a patient in cerebrovascular disease group and *R* denotes a patient in the reference group.

1 $$ CV=\left|\frac{BM{I}_C- BM{I}_R}{BM{I}_R}\right|+3\times \left|\frac{Ag{e}_C- Ag{e}_R}{Ag{e}_R}\right|, $$

The matching was started from the oldest patient with a cerebrovascular disease, as there was a lack of very old patients in the reference population.

Cardiorespiratory polygraphic recordings of stroke and TIA patients were conducted with Embla N7000 (Embla Systems LCC, Broomfield, CO, USA) at KUH. The recordings of the reference patients were ambulatory polygraphic studies obtained using Embletta (Natus Medical Inc., CA, USA) or Venla [16] device at patient’s home. Venla and Embletta have been shown to have similar diagnostic capabilities and there are no statistically significant differences between the devices in their estimation of AHI or ODI [16]. Notable difference between the devices is that abdomen and thorax movements are recorded with strain gauge in Venla and piezo belts in Embletta [17]. All recordings were analyzed based on blood oxygen saturation, nasal pressure, oronasal thermistor, respiratory effort, body position, and snoring signals using RemLogic (version 3.2, Embla Systems LCC, Broomfield, CO, USA) software and by following scoring criteria defined by American Academy of Sleep Medicine (AASM) in 2012 [7]. After the automated pre-analysis, used to achieve an initial analysis of the respiratory events, the apnea, hypopnea, and desaturation events were manually checked to ensure that the events and their durations were correctly detected. An apnea event was scored if the amplitude of the oronasal thermistor signal decreased by ≥ 90% from the pre-event baseline for a period of ≥ 10 s. A hypopnea event was scored if the amplitude of the airflow signal decreased ≥ 30% from the pre-event baseline for a period of ≥ 10 s, followed by ≥ 3% drop in the oxygen saturation signal. The AASM 2012 scoring criteria state that a hypopnea can also be scored without an oxygen desaturation, if the event is followed by an arousal defined from the EEG signal [7]. In this study, EEG signals were not recorded for the reference population and therefore the EEG signals were not used in the scoring of either of the groups. Desaturation events were determined to start from the first sampling point where the saturation signal started to decrease and to end at the last sampling point prior to the rise of the saturation.

Apnea-hypopnea index (AHI), apnea index (AI), hypopnea index (HI), and oxygen desaturation index (ODI) were calculated for each patient. Furthermore, the durations of each individual obstruction and desaturation event were calculated. Calculations were made with custom-made Matlab (version R2013b, MathWorks Inc., Natick, MA, USA) functions. The parameters and their definitions for individual event severity (ObsSev) are presented in Eqs. 2 and 3.

2 $$ Individual\kern0.1cm apnea\kern0.1cm event\kern0.1cm severity\kern0.1cm \left({s}^2\%\right)= ApDu{r}_i\times DesAre{a}_i $$

3 $$ Individual\kern0.1cm hypopnea\kern0.1cm event\kern0.1cm severity\kern0.1cm \left({s}^2\%\right)= HypDu{r}_j\times DesAre{a}_j, $$

where apnea and hypopnea duration are denoted as ApDur and HypDur respectively and desaturation area is denoted as DesArea. The index of the event is denoted as *i* and *j*.

The stroke and TIA patients were examined in separate groups, as well as their corresponding reference population matches. Distributions of individual event data were formed by dividing *x*-axis of apnea, hypopnea, and desaturation duration to 3 s, desaturation area to 10 s%, desaturation depth to 1%, and individual apnea/hypopnea event severity to 500 s^2^% wide bins and normalizing the number of the events with the total analyzed time of the corresponding group. In addition, all analyses were also performed between cerebrovascular disease patients (i.e., stroke and TIA patients pooled together) and reference patients after excluding all non-OSA patients (AHI < 5) from both groups.

Statistical significance of differences in all conventional parameters as well as durations and severities of individual events were evaluated with Mann-Whitney U test. Statistical analyses were performed with SPSS software (version 23, IBM Corporation, NY, USA) and *p* < 0.05 was used as the limit for statistical significance.

## Results

Table 1 summarizes the characteristics of the study groups; 65% of the stroke patients and 43% of the TIA patients were male. Stroke and TIA patients were generally overweight, and their age range was wide. Reference patients had similar characteristics as the cerebrovascular disease patients according to their gender, age, and BMI. Stroke and TIA patients slept significantly more in a supine position than the reference patients (*p* < 0.001, Table 1). It must be noted that to ensure venous return, the stroke and TIA patients slept in semi-recumbent supine position with head of the bed elevated at 30°. The utilized measurement device (Embla N7000) interpreted the semi-recumbent supine and non-supine positions as it would without the head of the bed elevation.

**Table 1:** Medians (range) of the population characteristics and the conventional sleep apnea parameters

	Stroke	Stroke reference	*p* value	TIA	TIA reference	*p* value
Patients (*n*)	49	49		28	28	
Gender (*n*_male_)	32	32		12	12	
Age (years)	69.5 (34.2–88.3)	66.0 (34.5–79.3)	0.084	72.3 (31.5–88.0)	69.0 (30.8–79.2)	0.098
BMI (kg/m^2^)	28.3 (20.6–42.2)	28.7 (21.1–47.3)	0.597	26.3 (19.8–43.7)	27.6 (20.3–39.8)	0.138
AHI (1/h)	17.4 (2.2–84.7)	23.3 (3.5–68.0)	0.553	10.9 (2.4–57.1)	22.0 (1.0–54.0)	0.140
ODI (1/h)	15.7 (1.9–83.2)	22.1 (3.5–66.0)	0.369	10.1 (2.3–50.2)	21.9 (1.0–53.8)	0.128
AI (1/h)	4.2 (0.0–56.5)	3.6 (0.0–56.2)	0.346	1.4 (0.0–36.6)	3.1 (0.0–29.6)	0.237
HI (1/h)	11.5 (1.7–78.9)	15.0 (3.4–53.71)	0.049*	8.7 (1.8–29.0)	17.0 (0.7–43.7)	0.064
AI/HI ratio	0.35 (0.0–13.28)	0.19 (0.0–4.76)	0.091	0.13 (0.0–2.19)	0.19 (0.0–1.87)	0.869
Obstructive AI (1/h)	2.2 (0.0–49.5)	3.2 (0.0–44.3)	0.359	0.4 (0.0–15.4)	3.0 (0.0–26.7)	0.103
Mixed AI (1/h)	0.3 (0.0–40.8)	0.1 (0.0–15.5)	0.063	0.0 (0.0–15.7)	0.0 (0.0–17.2)	0.894
Central AI (1/h)	0.5 (0.0–26.1)	0.1 (0.0–14.3)	0.007*	0.1 (0.0–16.6)	0.0 (0.0–8.8)	0.174
Supine time (min)	300.8 (0.3–696.4) †	151.7 (0.0–462.2)	< 0.001*	331.7 (86.0–556.1) †	152.6 (22.3–571.9)	< 0.001*
Non-supine time (min)	219.0 (0.0–485.6) †	294.4 (0.0–639.1)	0.006*	209.4 (0.0–414.6) †	274.1 (0.0–557.6)	0.022*

*BMI* body mass index, *AHI* apnea-hypopnea index, *ODI* oxygen desaturation index, *AI* apnea index, *HI* hypopnea index, *TIA* transient ischemic attack

*Statistically significant (*p* < 0.05), evaluated using Mann-Whitney U test

†Stroke and TIA patients slept in a semi-recumbent position to ensure venous return

**Fig. 1:**
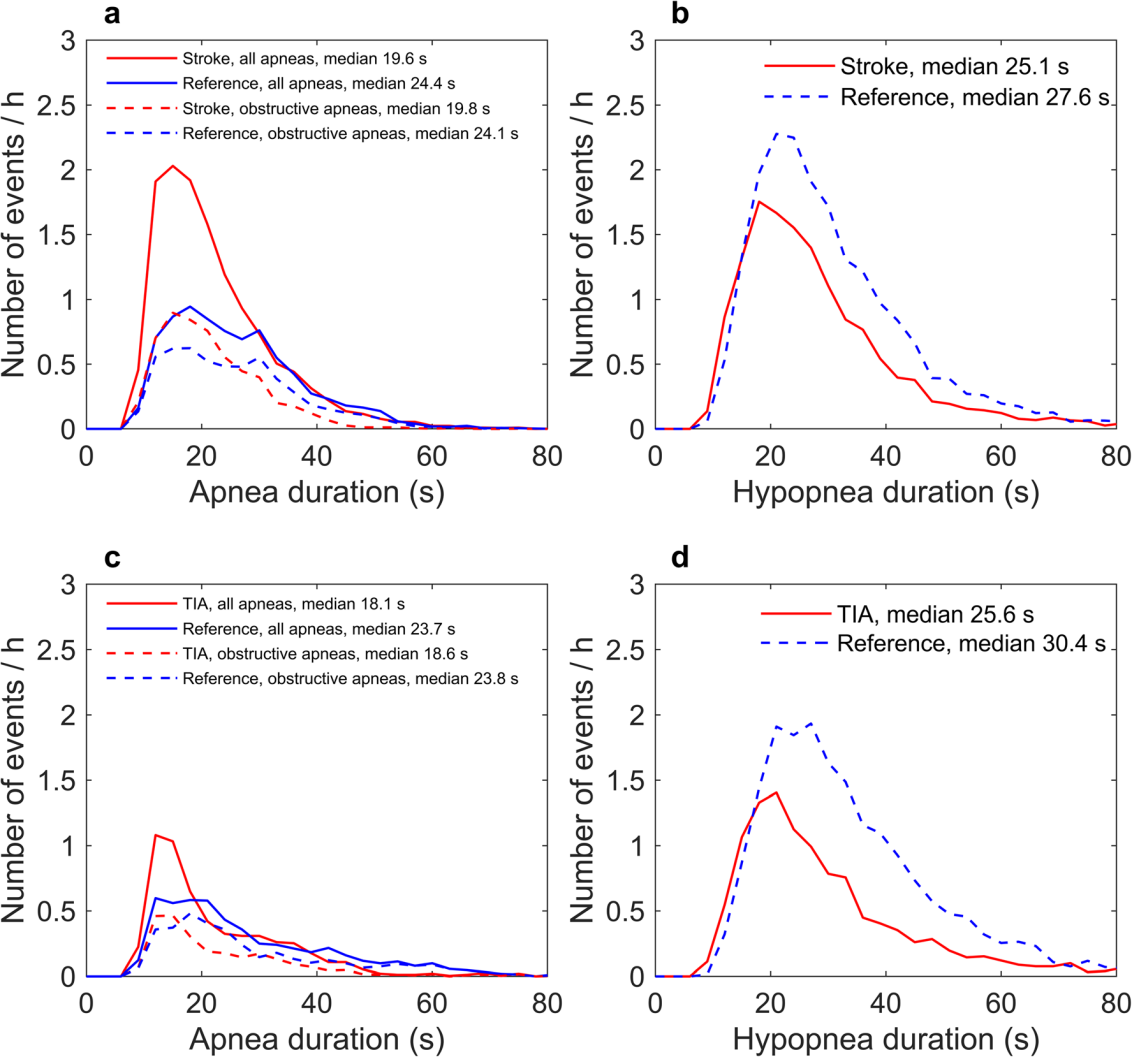
The distributions of individual apnea (mixed and central included, **a**, **c**), obstructive apnea (**a**, **c**) and hypopnea (**b**, **d**), durations in stroke (**a**, **b**) and TIA (**c**, **d**) patients, and their gender, age, and BMI-matched reference patients. The distributions are adjusted for the total analyzed time of the corresponding group

**Fig. 2:**
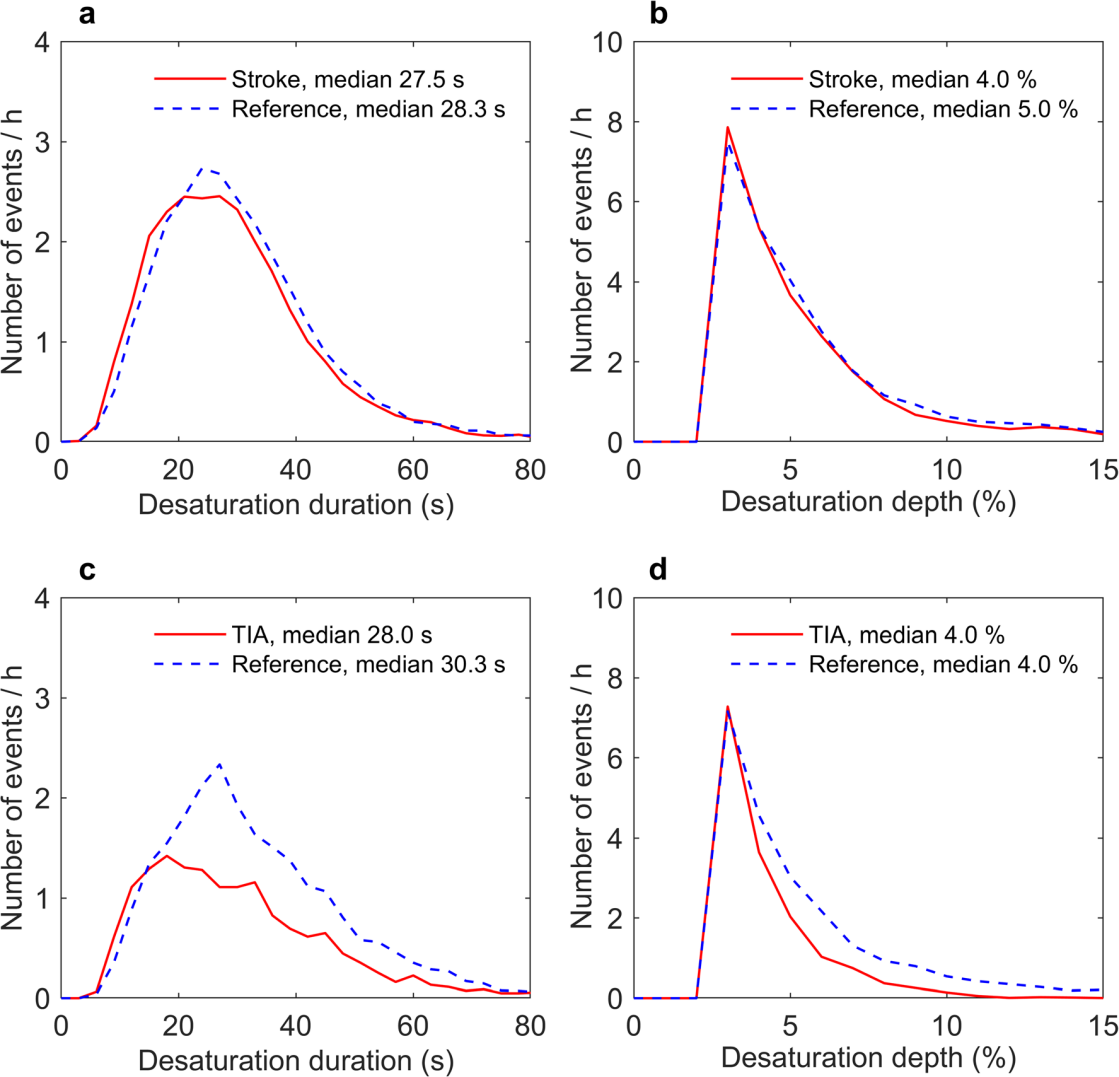
The distributions of individual desaturation event durations (**a**, **c**) and depths (**b**, **d**) in stroke (**a**, **b**) and TIA (**c**, **d**) patients and their gender, age, and BMI-matched reference patients. The distributions are adjusted for the total analyzed time of the corresponding group

**Fig. 3:**
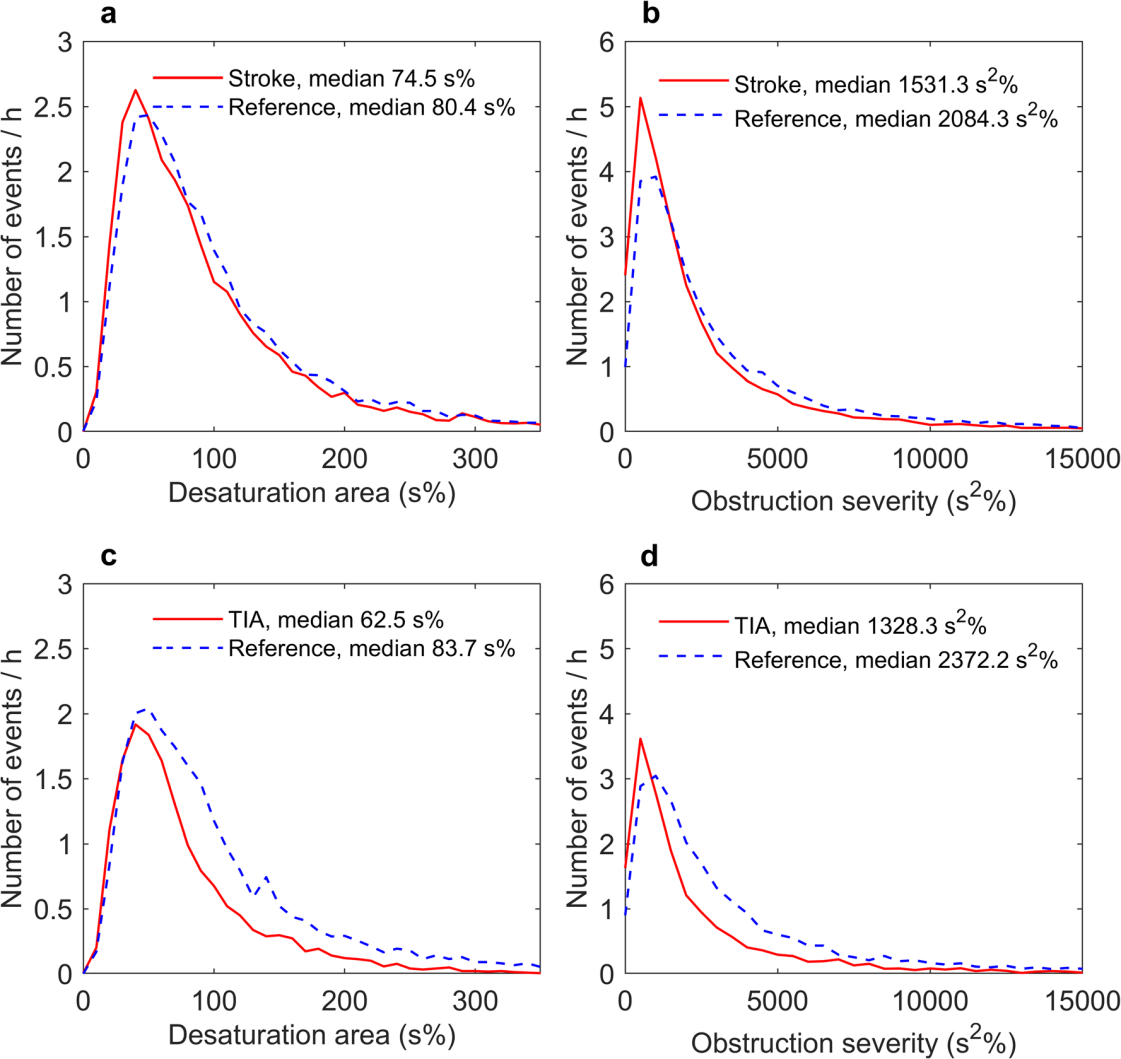
The distributions of desaturation area (**a**, **c**) and individual event severity (apneas and hypopneas combined, **b**, **d**) for individual events in TIA (**c**, **d**) and stroke patients (**a**, **b**) and their gender, age, and BMI-matched reference patients. The distributions are adjusted for total analyzed time of the corresponding group

Apnea and hypopnea events were significantly (*p* < 0.001) shorter in stroke and TIA patients than in the matched reference patients with suspected OSA (Table 2, Fig. 1). The shorter apnea and hypopnea events together with significantly (*p* < 0.001) smaller desaturation areas led to statistically significantly lower severities of individual apnea events (*p* < 0.001) as well as reduced individual hypopnea event severity. Based on visual inspection, the event distributions of the duration, depth, and area of desaturation events appeared similar in stroke and reference patients (Figs. 2 and 3), even though statistically significant differences were detectable in the duration and area of desaturation events. However, the difference is unlikely to be clinically significant as the test was overpowered due to the large event sample size (*n* > 4000). Furthermore, the event distributions revealed that TIA patients experienced fewer and shorter events than the reference patients (Figs. 1, 2, and 3).

**Table 2:** Medians (range) of the individual event parameters for stroke and TIA patients, and their matched reference patients

		Stroke	Stroke reference	*p* value*	TIA	TIA reference	*p* value*
Apnea	Duration (s)	19.6 (10.0–123.0)	24.4 (10.0–86.1)	< 0.001	18.1 (10.0–86.0)	23.7 (10.0–85.6)	< 0.001
	DesDur (s)	28.5 (4.0–119.5)	30.0 (5.7–97.3)	< 0.001	28.0 (6.5–95.5)	33.7 (7.0–88.0)	< 0.001
	DesArea (s%)	93.0 (5.5–2786.0)	112.0 (8.3–1344.7)	< 0.001	69.5 (2.0–1009.0)	134.2 (14.0–1390.3)	< 0.001
	ObsSev (s^2^%)	1788 (90–334,553)	2745 (88–115,709)	< 0.001	1385 (20–65,777)	3238 (148–116,204)	< 0.001
Hypopnea	Duration (s)	25.1 (10.0–193.4)	27.6 (10.0–254.8)	< 0.001	25.6 (10.0–176.1)	30.4 (10.0–157.9)	< 0.001
	DesDur (s)	27.0 (4.5–188.0)	27.7 (3.0–260.3)	< 0.001	28.0 (5.0–178.0)	29.7 (4.7–160.3)	< 0.001
	DesArea (s%)	64.5 (7.5–1426.0)	72.3 (3.0–1609.5)	< 0.001	60.0 (7.0–886.5)	77.0 (6.7–1338.3)	< 0.001
	ObsSev (s^2^%)	1619 (82–152,411)	1985 (49–372,108)	< 0.001	1508 (121–112,878)	1953 (83–115,860)	< 0.001

*DesDur* desaturation duration, *DesArea* desaturation area, *ObsSev* individual event severity (Eqs. 2 and 3)

*Statistical significance was evaluated with Mann-Whitney U test

Stroke patients had significantly higher central AI (*p* = 0.007) and a trend for higher mixed AI (*p* = 0.063) when compared to the reference patients (Table 1). In TIA patients, there was no statistically significant difference with respect to central or mixed AI as compared to the reference patients.

**Fig. 4:**
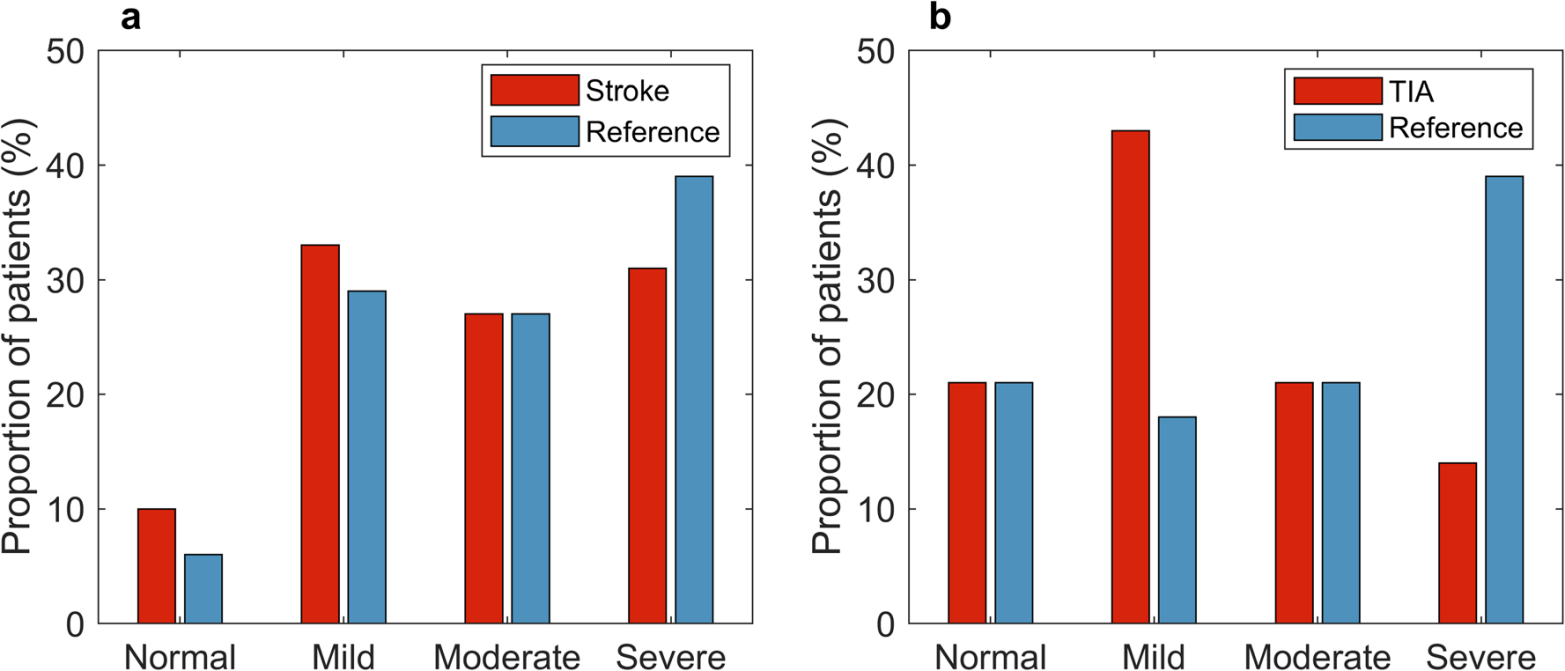
The proportion (%) of undiagnosed OSA in stroke (**a**) and TIA (**b**) patients, and diagnosed OSA in gender, age, and BMI-matched reference patients within different OSA severity categories

The prevalence of undiagnosed OSA (AHI ≥ 5) was 90% (44/49) in ischemic stroke patients and slightly less, i.e., 79% (22/28) in TIA patients (Fig. 4). In the reference groups matched by gender, age, and BMI with stroke and TIA patients, the prevalences of diagnosed OSA were 94% and 79%, respectively (Fig. 4). There was no statistically significant difference in the severity of OSA between stroke and TIA patients and their matched reference patients based on values of AHI and ODI. Stroke patients had a significantly lower median hypopnea index (*p* = 0.049), but median apnea index was not significantly different (*p* = 0.142) when compared with the reference patients (Table 1, Fig. 1). This led to a trend towards a higher AI/HI ratio in stroke patients as compared to the reference patients (0.35 vs. 0.19, *p* = 0.091). The apnea duration distributions (Fig. 1) indicated that a large portion of the apnea events in stroke and TIA patients are mixed or central apneas, even though the median values of mixed AI and central AI are low in both the stroke and TIA groups. According to the distributions, the total number of obstructive apnea events in stroke and TIA patients is similar to those present in the reference groups.

Results remained highly similar after excluding all non-OSA patients from the analyses. OSA patients with cerebrovascular disease had significantly lower HI compared to reference OSA patients without cerebrovascular disease (*p* = 0.002, Appendix Table 3). In addition, the trend for higher AI/HI ratio in patients with cerebrovascular disease than in reference patients was slightly stronger (*p* = 0.051, Appendix Table 3) compared to analyses performed separately to stroke and TIA patients. Patients with cerebrovascular disease had significantly shorter apnea, hypopnea, and desaturation durations, smaller desaturation areas, and lower severity of individual obstruction events (*p* < 0.001 for all parameters, Appendix Table 4). The median semi-recumbent supine and non-supine AHIs in the cerebrovascular disease patients were 20.5 h^−1^ and 5.9 h^−1^, respectively, which have statistically significant difference (*p* < 0.001).

## Discussion

Here, polygraphic recordings of 49 acute ischemic stroke patients and 28 TIA patients were compared to polygraphic recordings of gender, age, and BMI-matched reference patients suspected of having sleep-disordered breathing but no prior history of cerebrovascular disease. Along with conventional diagnostic parameters, severities of the individual apnea, hypopnea, and desaturations events were investigated.

Most importantly, we observed that the polygraphic phenotype of OSA is different in stroke and TIA patients when compared to reference patients without cerebrovascular disease. At odds with our hypothesis, the apnea, hypopnea, and desaturation events were shorter in patients with stroke or TIA than in the reference patients. This led also to significantly lower values of individual apnea event severity and individual hypopnea event severity in stroke and TIA patients as compared to reference patients. Highly similar results were obtained when non-OSA patients were excluded from the analyses and patients with cerebrovascular disease were considered as a single group. The results partly contradict with a previous study, which reported no significant difference in average apnea duration and longer maximal apnea duration in cerebrovascular disease patients compared with controls [2]. However, this previous study did not consider individual respiratory events.

Short events have previously been associated with increased all-cause mortality, which might connect short events to lower arousal threshold and elevated sympathetic tone [8]. Acute phase of stroke has been previously associated with deterioration of sleep quality [18, 19]. Possible low arousal threshold combined with light and fragmented sleep could at least partially explain the observed short event durations. In general, hypoxic burden measured as the sum of desaturation areas, has been shown to predict the cardiovascular disease–related mortality [11]. However, it is most likely that high level of hypoxia indicated by large desaturation areas, and low arousal threshold possibly indicated by short events, could both be associated to severe vascular consequences of OSA.

In the present study, ischemic stroke patients had significantly higher central AI, which is consistent with previous studies [20–22]. Previously, it has been shown that central sleep apnea is associated with stroke in elderly subjects, and that central sleep apnea could be a marker for a silent stroke [21]. In addition, acute stroke patients have higher probability for central apneas than other OSA patients [22]. Furthermore, it has been shown that the AHI decreases after the acute phase of stroke. This could be partially explained by a decrease in the numbers of central apnea events after the acute phase of stroke [20, 23].

There was no statistically significant difference in AHI between stroke patients and corresponding reference patients, or between TIA patients and their reference patients. Stroke patients had significantly lower HI (*p* = 0.049) and showed a trend towards a higher AI/HI ratio (*p* = 0.091). The proportion of apnea events was found to be higher in stroke patients than in the reference patients. On the other hand, stroke patients were found to have slightly lower AHI values and significantly shorter apnea and hypopnea events. Previously, it has been reported that obstructive apneas induce more severe desaturations than hypopneas [24]. According to the event duration distributions (Fig. 1), the relatively higher number of apnea events in stroke patients is not fully explained by the higher number of central apneas. Based on the present results, it seems that the polygraphic phenotype of OSA is different in acute stroke in comparison to a matched reference population. This is potentially an important finding with respect to the prognostics of cerebrovascular disease since untreated OSA has been shown to significantly worsen the rehabilitation outcomes of stroke and increase the risk for recurrent cardiovascular events [4–6]. However, it remains unclear whether the different polygraphic features are at least partially present before the stroke and part of the normal development of OSA or whether acute stroke and its treatment also modulate the phenotype of OSA. Therefore, the present results indicate need for further research.

The prevalence of OSA (AHI ≥ 5) was 90% in the acute ischemic stroke patients and 79% in the TIA patients. It is worthwhile noting that these stroke and TIA patients had no previous suspicion of OSA. In the reference groups matched with stroke and TIA patients, the prevalences of OSA were 94% and 79%, respectively. This result is consistent with previous studies describing a high OSA prevalence in stroke and TIA patients. In a recent meta-analysis of 3242 ischemic stroke, hemorrhagic stroke, and TIA patients, the prevalence of OSA was estimated to be 70.4% [3]. The higher prevalence of OSA in the present study may be partly caused by the use of a lower 3% oxygen desaturation limit for scoring of hypopneas. In addition, stroke and TIA patients were analyzed separately in the present study, which was not performed in the meta-analysis [3]. Thus, the high prevalence of OSA in stroke and especially in TIA patients is a clinically significant finding as OSA has been linked to a higher probability of recurrence of cerebrovascular incidents [25] as well as a risk of death [26]. The present findings, together with reports in the literature, support the idea of screening for OSA in all stroke and TIA patients. This introduces a need for simplistic and cost-effective routine screening methods for sleep-disordered breathing.

This study is not without limitations. The analyzed signals did not include EEG, and therefore the sleep stages and hypopneas related only to cortical arousals could not be scored. Furthermore, the total sleep time was determined purely based on the polygraphic signals. Despite these shortcomings of polygraphic recordings, AASM has stated that unattended portable monitors not including EEG registration are reliable as diagnostic tools for OSA [27]. Furthermore, polygraphy recordings have been shown to have sufficient accuracy in estimating severity of OSA in acute in-hospital stroke patients [28], and high accuracy in discriminating central and obstructive apnea in patients with heart failure [29]. The recordings of the stroke and TIA patients were performed with Embla N7000; those from the reference populations were recorded using portable monitors (either Embletta or Venla). Embletta and Venla have been shown to have similar diagnostic capabilities and there are no significant differences in the values of the conventional diagnostic parameters (e.g., AHI, ODI) [16]. Embletta and Embla devices are manufactured by the same company, Embla Systems LCC. Therefore, the use of three different devices should not influence the validity of the present results.

We acknowledge that the relatively low number of patients is a major limitation in this study and therefore statistical significance was not always reached even though there were evident differences in median values between the groups. However, the primary objective of this study was to investigate the difference in the severity of individual events between patients with and without cerebrovascular disease. Therefore, even with a relatively low number of patients, the total number of events was more than sufficient (*n*_*min*_ = 2463, *n*_*max*_ = 11,020, depending on the calculated parameter) to permit a relevant statistical analysis. Furthermore, location and severity of ischemic stroke was not taken into account. However, the stroke patients examined in this study represent well the patients treated in Kuopio University Hospital.

In the current clinical practice in Finland, acute stroke patients are positioned with the head of the bed elevated at 30 degrees to ensure the venous return. This may have exerted a slight impact on the observed OSA severity as it has been shown that even a mild elevation of 7.5° reduces OSA severity in both supine and non-supine position [30], and a seated position increases the upper airway area [31] and decreases the upper airway collapsibility [32]. However, as stated above, semi-recumbent position in acute stroke patients is a standard procedure. Therefore, the results obtained in this study represent the actual sleeping positions and polygraphic phenotype of OSA during the treatment after acute phase of stroke. Despite this semi-recumbent position, definite OSA is still present and poses the patients to significant risks, impairs the recovery from stroke, and thereby requires active OSA treatment.

Stroke and TIA patients slept more in the semi-recumbent supine position than the reference patients in the supine position. A high proportion of semi-recumbent supine sleep in stroke patients is in line with a previous study [33]. This may be due to the partial immobility caused by stroke, elevated head of the bed, and sleeping in a neurological ward connected to a more complex polysomnographic recording device. High proportion of supine sleep has a direct increasing influence on AHI as it has been shown that apneas and hypopneas are more common when an individual is sleeping in the supine position [34]. Supine sleep remains unfavorable compared to non-supine when the head of the bed is elevated. In the stroke patients recruited for the present study, the median semi-recumbent supine and non-supine AHIs were 20.5 h^−1^ and 5.9 h^−1^, respectively (*p* < 0.001). This indicates that positional therapy where patient is prevented from sleeping on his/her back might be a useful option for reducing OSA severity in many post-stroke patients.

Screening for OSA after acute cerebrovascular disease is not performed in a routine basis. We found that patients with acute cerebrovascular disease had shorter apnea and hypopnea events compared to reference outpatient population. The observed event characteristics might indicate that patients with acute cerebrovascular disease suffer from OSA phenotype recently associated with increased cardiovascular mortality. Therefore, optimal OSA diagnostics and subsequent treatment require routine OSA screening in patients with acute cerebrovascular disease, even without previous clinical suspicion of OSA.

## Electronic supplementary material

ESM 1 (PNG 684 kb)

High resolution image (TIF 8508 kb)

## Appendix

**Table 3:** Medians (range) of the population characteristics and the conventional diagnostic parameters when stroke and TIA populations are pooled together and non-OSA (AHI < 5) patients are excluded from the analysis

	Cerebrovascular disease patients	Reference patients	*p* value
Patients (*n*)	66	68	
Gender (*n*_male_)	29	31	
Age (years)	70.7 (45.4–88.3)	67.3 (48.5–79.3)	0.018*
BMI (kg/m^2^)	27.4 (19.8–43.7)	27.8 (20.3–47.3)	0.343
AHI (1/h)	18.4 (5.8–84.7)	24.1 (5.7–68.0)	0.130
ODI (1/h)	16.1 (4.9–83.2)	23.3 (5.7–65.9)	0.055
AI (1/h)	4.1 (0.0–56.5)	4.3 (0.0–56.2)	0.598
HI (1/h)	12.3 (2.9–78.9)	18.9 (4.5–53.7)	0.002*
AI/HI ratio	0.37 (0.0–13.28)	0.21 (0.0–4.76)	0.051
Obstructive AI (1/h)	2.3 (0.0–49.4)	3.5 (0.0–44.3)	0.251
Mixed AI (1/h)	0.3 (0.0–41.0)	0.1 (0.0–17.2)	0.054
Central AI (1/h)	0.5 (0.0–26.1)	0.1 (0.0–14.3)	0.003*
Supine Time (min)	331.7 (0.3–696.4) †	148.1 (0.0–571.9)	< 0.001*
Non-supine Time (min)	210.2 (0.0–485.6) †	292.55 (0.0–639.1)	< 0.001*

*BMI* body mass index, *AHI* apnea-hypopnea index, *ODI* oxygen desaturation index, *AI* apnea index, *HI* hypopnea index, *TIA* transient ischemic attack

*Statistically significant (*p* < 0.05), evaluated using Mann-Whitney U test

†Stroke and TIA patients slept in a semi-recumbent position to ensure venous return

**Table 4:** Medians (range) of the individual event parameters for OSA patients with cerebrovascular disease (stroke or TIA) and reference OSA patients without cerebrovascular disease

		Cerebrovascular disease patients	Reference patients	*p* value*
Apnea	Duration (s)	19.4 (10.0–123.0)	24.3 (10.0–86.1)	< 0.001
	DesDur (s)	25.5 (4.0–119.5)	29.6 (5.7–97.3)	< 0.001
	DesArea (s%)	87.5 (2.0–2786.0)	117.0 (8.3–1390.3)	< 0.001
	ObsSev (s^2^%)	1707 (20–334,553)	2886 (88–116,204)	< 0.001
Hypopnea	Duration (s)	25.1 (10.0–193.4)	28.6 (10.0–254.8)	< 0.001
	DesDur (s)	27.0 (4.5–118.0)	28.3 (4.3–260.3)	< 0.001
	DesArea (s%)	63.0 (7.0–1426.0)	74.0 (3.0–1609.5)	< 0.001
	ObsSev (s^2^%)	1578 (82–152,411)	2092 (48–372,108)	< 0.001

*DesDur* desaturation duration, *DesArea* desaturation area, *ObsSev* individual event severity (Eqs. 2 and 3)

*Statistical significance was evaluated with Mann-Whitney U test
