# Supplementary figures and images for: Acute stroke and TIA patients have specific polygraphic features of obstructive sleep apnea
Source: Sleep Breath. 2020 Jan 14;24(4):1495–505. doi: 10.1007/s11325-019-02010-2 (PMC7679322; doi:10.1007/s11325-019-02010-2)

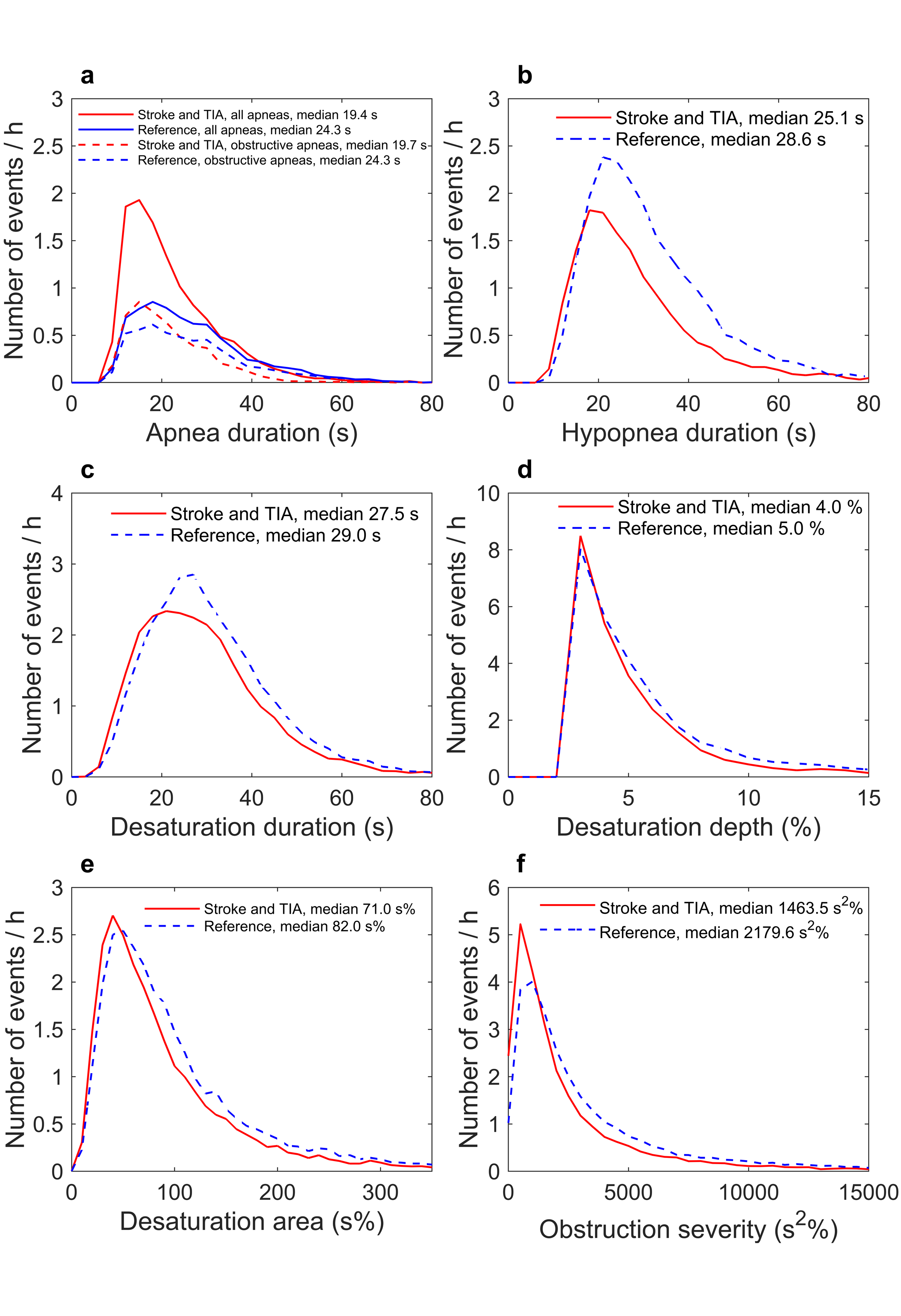

Supplement: Supplementary file 1 — (PNG 684 kb) [file 11325_2019_2010_Fig5_ESM.png]
